# Analysis of rare coding variants in schizophrenia-associated genes and generalised cognition in the UK Biobank

**DOI:** 10.1038/s41380-026-03601-8

**Published:** 2026-04-10

**Authors:** Eilidh Fenner, Peter Holmans, Michael C. O’Donovan, Michael J. Owen, James T. R. Walters, Elliott Rees

**Affiliations:** Centre for Neuropsychiatric Genetics and Genomics, Division of Psychological Medicine and Clinical Neurosciences, Cardiff University, Cardiff, UK

**Keywords:** Schizophrenia, Genetics

## Abstract

Cognitive impairments in schizophrenia are associated with poor outcomes and are largely unimproved by current medications. It remains uncertain to what extent cognitive impairments arise from shared aetiology and biology with schizophrenia, or are a consequence of having the condition. We analysed exome-sequencing data from 76,783 UK Biobank participants without schizophrenia to test for association between generalised cognition (*g*) and rare (minor allele count ≤ 5) variants in schizophrenia-associated genes. Protein-truncating and deleterious missense variants in loss-of-function intolerant genes were associated with lower *g*. Significantly stronger effects on *g* were found for protein-truncating variants in genes implicated in schizophrenia by rare coding variation, and for deleterious missense variants in credible causal genes at schizophrenia common allele loci. These findings indicate that biological processes disrupted in schizophrenia by common and rare variants are associated with *g* in unaffected individuals, suggesting the relationship between impaired cognition and schizophrenia reflects, in part, a shared underlying biology.

## Introduction

Schizophrenia is a severe and clinically heterogeneous psychiatric disorder with a lifetime prevalence of just under 1% [1, 2]. Impaired cognitive function is an important feature of schizophrenia that often precedes the onset of psychosis [3–5] and is a strong predictor of functional outcomes, encompassing social, occupational, educational, and daily functioning [6, 7]. Relative to controls, those with schizophrenia demonstrate impairments across multiple cognitive domains, indicating a generalised cognitive impairment [8].

Generalised cognition can be represented by *g*, a psychometric construct representing a latent variable that underlies performance across diverse cognitive tests and domains [9]. It is derived using factor analysis or principal component analysis of a range of cognitive measures, and it thus reflects the shared variance across these measures [10–12]. Although the derivation may vary across studies, the resulting factors are highly correlated with one another and with measures of IQ, and consistently capture the same underlying construct of generalised cognitive ability [10, 11, 13, 14]. This study focuses on *g*, instead of individual cognitive tests, as schizophrenia is associated with a generalised rather than primarily domain-specific cognitive deficit [8, 15, 16]. Measures of *g* in individuals with schizophrenia are on average around 1.5 standard deviations lower than in controls [17–19]. Moreover, *g* explains the majority of variance in cognition between people with schizophrenia and healthy controls, with two thirds of the overall effect of a schizophrenia diagnosis on cognition being mediated through *g* [8] and minimal domain-specific effects once *g* has been accounted for [20]. *g* is also a better predictor of functional outcome in schizophrenia than any individual cognitive test [21].

Schizophrenia is highly heritable and polygenic, with liability conferred by rare and common alleles in many genes, particularly those under selective constraint against mutations predicted to result in a loss of protein function, also known as loss-of-function intolerant (LoFi) genes [22–24]. The largest genome-wide association study (GWAS) of common alleles (minor allele frequency (MAF) > 1%) in schizophrenia to date identified 287 distinct associated loci [25], and among these, fine-mapping prioritised 106 protein-coding genes as credibly causal. The genome-wide burden of rare copy number variants (CNVs) is also increased in schizophrenia cases compared with controls, with a set of 63 CNVs reported to be associated with developmental disorders showing particular enrichment in cases [26, 27]. Moreover, 13 of these CNVs have been robustly associated with schizophrenia liability [26, 27]. Finally, sequencing studies have identified 12 genes that are individually enriched in schizophrenia for damaging types of rare coding variants (RCVs) [28, 29]. There is also evidence for a convergence between genes implicated in schizophrenia by common and rare alleles [25, 28].

Genetic liability for schizophrenia is pleiotropic, with shared effects on liability to several other psychiatric and developmental disorders as well as on variation in cognitive function in the general population. For example, the 13 CNVs robustly associated with schizophrenia are all risk factors for developmental disorders [26], and individuals without a psychiatric or developmental disorder who carry one of these CNVs perform worse on a range of cognitive tests compared to non-carriers [30, 31]. Similarly, damaging RCVs in developmental disorder-associated genes are associated with poorer cognition in individuals without a psychiatric or developmental disorder [32, 33]. Finally, a higher burden of schizophrenia common alleles is associated with lower cognitive function in the population [34, 35].

Less is known about the effects of damaging RCVs in schizophrenia genes and loci on cognition in individuals without a diagnosis of schizophrenia, autism spectrum disorder (ASD) or intellectual disability (ID). Studying these individuals helps isolate the direct effects of rare variant liability for schizophrenia on cognition, independent of the effects of other factors related to the pathophysiology of the disorder, or that emerge as a consequence of schizophrenia or other neurodevelopmental disorders (e.g. antipsychotic medication use, long-term symptoms, or lifestyle factors). This reduces the risk of reverse causation, whereby differences in cognitive ability may be a consequence of the disorder and/or its treatment. It is therefore important to establish whether damaging RCVs in schizophrenia-associated genes impact cognition in those unaffected by this disorder, as this would provide evidence that the association of impaired cognition with schizophrenia is, at least in part, explained by shared biology between schizophrenia and cognitive function.

Recent studies using data from the UK Biobank (UKBB) have shown associations of damaging RCVs in LoFi genes with longer reaction-times, lower fluid intelligence scores, and lower levels of educational attainment [32, 36]. Similar associations were observed for damaging RCVs in genes mapping to schizophrenia GWAS loci, however, it remains unclear whether this association was attributable to those loci being associated with schizophrenia, or whether it was confounded by the fact that LoFi and brain-expressed genes are enriched within these schizophrenia loci [24, 32]. It also did not test the specific genes within those loci that have been reported to be credibly causal. There has also been no study examining the relationship between cognition in individuals without a psychiatric or developmental disorder and RCVs in sets of genes implicated in schizophrenia by rare CNVs or RCVs.

In the current study, we analysed whole exome sequencing (WES) data from 396,848 unrelated UKBB participants passing quality control (QC) to examine whether damaging RCVs in genes implicated in schizophrenia have effects on cognition in people without a diagnosis of schizophrenia, ASD, or ID (full study design presented in Methods, summary presented in Supplementary Figure 1). Whereas previous UKBB RCV studies of cognition analysed only individual cognitive tests [32, 33], we analysed a measure of *g*, derived from a principal component (PC) analysis of four cognitive tests. In UKBB participants that are genetically similar to the 1000 Genomes Project (1KGP) European superpopulation, we demonstrate that rare protein-truncating variants (PTVs) and damaging missense variants in LoFi genes are associated with lower *g*, which to our knowledge has not been shown before. In these participants, we also show that rare PTVs in genes implicated in schizophrenia by RCVs have a significantly stronger effect on *g* than that observed for rare PTVs in LoFi genes and brain-expressed genes. Additionally, rare deleterious missense variants in credible causal genes underlying schizophrenia common allele loci were associated with lower *g*, and this effect was significantly stronger than that observed for rare deleterious missense variants in all genes mapping to the schizophrenia common allele loci, and for rare deleterious missense variants in LoFi genes or brain-expressed genes.

Overall, these findings indicate that biological processes disrupted in schizophrenia by common and rare variants are linked to *g* in unaffected individuals. This suggests the association of impaired cognition with schizophrenia reflects shared underlying biology between schizophrenia and cognitive function.

## Methods

### UK Biobank

Between 2006 and 2010, the UKBB recruited around 500,000 participants from the UK through NHS registers, with no exclusion criteria apart from reasonable access to an assessment centre. Participants were aged 40–69 at recruitment and 54% were female. All participants gave informed consent for their data to be used by UKBB projects and agreed to being followed up. This research was conducted using the UKBB resource under Application 13310.

### Array data

UKBB participants were genotyped using the Affymetrix Axiom UKBB array (~450,000 individuals) and the UK BiLEVE array (~50,000 individuals). Using this data, schizophrenia polygenic risk scores (PRS) were derived via a clumping and thresholding approach in PRSice-2 [37] using de-duplicated summary statistics from the largest GWAS of schizophrenia to date [25], as described in our previous publications [38, 39]. Array data was also used to identify carriers of CNVs known to be enriched in schizophrenia, which includes a set of developmental disorder (DD) associated CNV loci [26]. These CNVs were called in our previous publication [30, 40].

### Whole exome sequencing data

WES data for 469,385 UKBB participants were generated using the IDT xGen Exome Research Panel v1.0 exome capture kit and Illumina NovaSeq 6000 instruments; S2 flow cells were used for the initial 50k sequencing release, and S4 flow cells used for all following samples. Further details on sequencing protocol can be found in the primary UKBB publication [41].

#### Data processing and quality control

WES data was accessed and analysed using the UKBB Research Analysis Platform (RAP). Data was initially accessed as a joint-genotyped variant call set in pVCF format, which was centrally generated by the UKBB with Deep Variant 0.010 [42] using CRAMs that were processed using the ‘OQFE protocol’ [43–46], with reads aligned to GRCh38 reference genome. For further details, see [47, 48].

We performed genotype, sample, and variant QC using Hail (version 0.2.60 [49]) running on a JupyterLab instance on the RAP, and R (using RStudio v2.2.1) running on a Posit Workbench on the RAP. The original dataset spanned 22,854,479 variants and 469,385 individuals. Multiple steps of variant- and genotype-level QC were undertaken to ensure only high-quality variants remained in the dataset. The number of variants excluded and retained following each QC step are presented in Supplementary Table 1. Genotypes were excluded if they met any of the following criteria: depth ≤ 10x; genotype quality ≤ 30; homozygous genotypes for the reference allele with an allele balance > 0.1; homozygous genotypes for the alternate allele with an allele balance < 0.9; heterozygous genotypes with an allele balance < 0.25 or > 0.75. Variant sites were excluded if they met any of the following criteria: labelled as ‘mono-allelic’ [50]; > 6 alternative alleles (including indels and SNVs); no alternative alleles after applying genotype QC; within the X or Y chromosome; mean genotype quality across all samples ≤ 40; call rate ≤ 0.9; or overlapped a low-complexity region. In total, 19,724,617 variants remained following variant QC.

Sample level QC was undertaken to ensure only samples with high-quality sequencing data remained in the dataset. The number of samples excluded and retained following each QC step are presented in Supplementary Table 2. Participants were excluded if they met any of the following criteria: sequencing call rate < 0.8 (*n* = 234, Supplementary Figure 2); discordant self-reported sex, imputed sex, and Y chromosome depth (*n* = 210, Supplementary Figure 3, see Supplementary Materials 1.1 for detail); diagnoses of schizophrenia, ASD, or ID from primary care data, hospital inpatient data, death register records, or self-report (*n* = 1526); or insufficient data for inferring ancestries (lacking or low quality array data, *n* = 1293). No sample was excluded for low mean sequencing depth or mean genotype quality, most likely because this dataset had already received central QC prior to public release. To identify related individuals, we used estimates of pair-wise kinship co-efficients from array data. The ‘*ukb_gen_samples_to_remove’* command from the R package ukbtools [51] was used to remove 69,274 samples ensuring that no pairs of retained participants were third degree relations or closer, defined as a kinship co-efficient > 0.0442 [52, 53]. In total, 72,537 individuals failed sample QC, retaining 396,848 individuals for analysis.

Analyses for inferring genetic ancestry are based on the procedure described in [54] and updated in [55], and were conducted using UKBB array data, as in other UKBB exome sequencing studies [56, 57]. A description of this process is provided in detail in Supplementary Materials 1.2. Briefly, array data was used alongside biogeographical categories based on a standardised system [58] and a 1KGP Phase 3 reference [59] to infer Ancestry Informative Markers. PC-air was used to compute PCs in the 1KGP reference population, and project UKBB samples into the reference PC co-ordinates. The Tracy-Widom test [60] selected eigenvectors for ancestry inference and a model based on Fisher’s Linear Discriminant Analysis [61] was trained on known ancestries of the 1KGP reference samples, and then applied to the UKBB dataset to predict genetic ancestry probabilities. Individuals were assigned a 1KGP-like genetic ancestry label if any ancestry probability was > 0.8, and otherwise were assigned an ‘Admixed’ label. We note that these labels merely reflect genetic similarity to the 1KGP reference panel [59] and do not represent discrete or biologically distinct categories.

#### Variant annotation

RCVs, comprising single-nucleotide variants and insertions and deletions < 50 base-pairs in length, were annotated in Hail using Ensembl’s Variant Effect Predictor (VEP) [62]. We defined rare variants as those with a minor allele count (MAC) ≤ 5 (equating to a MAF < 6.25 × 10^−6^), a definition similar to that of other UKBB rare variant studies [32, 33, 56]. PTVs were defined as splice acceptor, splice donor, stop-gain or frameshift variants that were annotated as high confidence for causing loss of protein function by LoFTEE [63, 64]. Deleterious missense variants were defined as missense variants with a REVEL [65] score > 0.75. This REVEL score threshold was chosen based on specificity for predicting deleterious variants in the Human Gene Mutation Database [65]. We also examined rare synonymous variants as a negative control.

#### Schizophrenia-associated gene sets

All gene sets are described in detail in Supplementary Table 3. Genes under selective constraint for PTVs, known as LoFi genes, are enriched for damaging RCVs, rare CNVs, and common alleles in schizophrenia [22–24]. We therefore analysed RCVs in 3162 autosomal LoFi genes (defined as genes with a GnomAD pLI score [66] ≥ 0.9) and in the remaining LoF-tolerant genes (those with a GnomAD pLI score < 0.9). Many schizophrenia-associated gene sets are enriched for genes expressed in the brain. We therefore investigated RCVs in brain expressed genes, defined as genes with an average of > 5 fragments per kilobase of exon model per million mapped reads in the brain in an RNA sequencing study [67].

To provide robust evidence that the biology of schizophrenia overlaps with that for cognitive function, we also tested the following sets of protein-coding, autosomal genes implicated in schizophrenia in the largest GWAS [25], CNV [26] and RCV [28] studies of the disorder to date.

##### RCV enriched genes

Genes enriched for RCVs in schizophrenia were taken from the Schizophrenia Exome Sequencing Meta-Analysis Consortium (SCHEMA) study [28]. We analysed 29 autosomal genes enriched for RCVs in schizophrenia at a false discovery rate (FDR) of < 5%.

##### Common allele loci

Genes implicated in schizophrenia by common alleles were taken from the largest schizophrenia GWAS [25]. This study identified 282 autosomal loci associated with schizophrenia and prioritised credible causal protein-coding genes for some of these loci using fine-mapping and SMR supplemented by chromatin conformation analysis. We analysed three main schizophrenia common allele loci gene sets: **1)** all 1715 genes that overlapped the 282 implicated loci; **2)** 186 genes closest to the index SNP for each associated locus (loci where the nearest gene to the index SNP was non-coding were not included in this analysis); **3**) 101 credible causal protein-coding genes that were prioritised in the original paper [25].

##### Schizophrenia CNV loci

A set of 63 CNVs for which there is at least some evidence for association with developmental disorders have been shown to be collectively enriched in people with schizophrenia [26]. We investigated RCVs within 886 genes in regions defined by these 63 CNVs, that we refer to as the schizophrenia-enriched CNV gene set. 13 of these 63 CNVs have individually been associated with schizophrenia with robust statistical significance [26, 27], and we refer to the 178 genes within these loci as the schizophrenia-associated CNV gene set. To refine which genes within the schizophrenia-enriched CNV gene set may be contributing to *g*, additional exploratory analyses were performed by testing RCVs only in the schizophrenia-associated CNV gene set, and also by testing LoFi and LoF-tolerant genes in the schizophrenia-enriched and schizophrenia-associated CNV gene sets separately (see Supplementary Table 3 for further details).

### Phenotypic data

Phenotypic data were collected from UKBB participants at multiple timepoints, in person and online. All participants attended an initial assessment centre where baseline data were collected by: touchscreen questionnaires, including cognitive measures which took around 5 min; a face-to-face interview; and blood sample collection. Participants were later invited to attend additional visits and to complete online questionnaires, including online cognitive test batteries. Where cognitive tests were completed at multiple timepoints, we selected the instance with the greatest sample size for use in our study. Supplementary Table 4 provides an overview of the cognitive tests examined in this study and the number of participants who completed them. For all tests, scores were converted to a normal distribution if not already normally distributed and standardised through conversion to z-scores. Outlying scores (outside of +\- 4 standard deviations of the mean) were excluded for each cognitive test.

#### Generalised cognition

Cognitive assessments were brief and unsupervised. Individual cognitive tests are known to vary in their reliability and stability [68, 69], but performance between different cognitive tests is correlated [68, 70] (Supplementary Table 5). The latter property enables *g* to be derived from a PC analysis of multiple cognitive tests [69]. We derived *g* as the first PC from a PC analysis of the following: **1)** Numeric memory (online test battery 1), **2)** Reaction time, **3)** Pairs matching, **4)** Trail making test B (online test battery 1). Detailed descriptions of how *g* was derived, and the rationale for the inclusion/exclusion of individual cognitive tests in *g*, are provided in Supplementary Materials 1.3. In total, we had sufficient data to calculate *g* for 76,783 participants.

### Statistical analysis

Linear regression was used to test for association between *g*, the dependent variable, and RCV burden in different schizophrenia-associated gene sets and individual genes. We included as covariates burden of synonymous variants (apart from when investigating synonymous variant burden), sequencing batch and assessment centre. Given cognitive ability is non-linearly associated with age, and that this can differ by sex, we also included covariates for sex and standardised age, standardised age^2^, sex*standardised age and sex* standardised age^2^. Additionally, we included covariates to control for population stratification, including ancestry probabilities (excluding 1KGP-EUR-like probability, as if all ancestry probabilities were included these would be co-linear). We also covaried for relevant within-ancestry PCs 1–10 (e.g. for analysis of the 1KGP-EUR-like population, the first 10 PCs derived from just this population were included as covariates). For the analysis of the participants who were assigned the ‘Admixed’ 1KGP-like genetic ancestry label (see Supplementary Materials 1.2), we covaried for PCs derived in all participants rather than for within-ancestry PCs. We explored whether associations of RCV burden and *g* were explained by variation in specific cognitive tests by including individual cognitive test scores as covariates in the analyses of LoFi genes. All reported regression p-values are two-sided.

A multivariable linear regression model was used to test whether damaging RCVs, schizophrenia PRS, and the rare DD CNVs known to be enriched in schizophrenia (CNVs described in the ‘Array data’ section of the Methods) had independent effects on cognition. Here, we included all covariates used in the analysis of RCVs alone, as well as an additional covariate reflecting genotyping array (Affymetrix Axiom UKBB or UK BiLEVE array) and genotyping batch to account for potential technical genotyping differences [71]. This analysis was run in 75,111 individuals of 1KGP-EUR-like ancestries in whom *g* could be derived and who had high-quality data for all classes of genetic variation investigated.

To compare associations of RCV burden and *g* between gene sets, we performed z-tests using:

$$\frac{({\beta }_{1}-\,{\beta }_{2})}{\sqrt{{({\sigma }_{1})}^{2}+{({\sigma }_{2})}^{2}}}$$

where$\,{\beta }_{1}$ is the beta and ${\sigma }_{1}$ is the standard error of association from the RCV burden in gene set 1, and ${\beta }_{2}$ is the beta and ${\sigma }_{2}$ is the standard error of association from the RCV burden in gene set 2. Only independent gene sets were compared using z-tests.

## Results

### Distribution of protein-coding variation

19,724,617 high-quality autosomal protein coding variants were observed in 396,848 unrelated UKBB participants that passed QC. 75.6% of these variants were rare (MAC ≤ 5). Substantial variation was observed in the number of rare variants per participant across 1KGP-like genetic ancestries (Supplementary Table 6), with participants that were genetically similar to the 1KGP European superpopulation (*n* = 376,728) carrying fewer rare damaging variants (mean number of rare PTVs carried = 1.78) compared with participants genetically similar to other 1KGP superpopulations (mean number of rare PTVs carried ranging from 4.81 to 6.97; Supplementary Table 6). This difference was expected given allele frequencies were defined by the UKBB sample itself, and 95% of the cohort were of 1KGP-EUR-like genetic ancestry. We controlled for differences in the rates of RCVs across populations by analysing participants in each genetically inferred ancestry group separately.

### Estimating generalised cognition in the UKBB

In the 76,783 participants in whom *g* could be derived, *g* explained 38.9% of the variance of the tests included in its formation (numeric memory, reaction time, pairs matching, and trail making test B; see Supplementary Table 4 for a description of these tests). As expected*, g* was moderately correlated with each of the individual tests used in its derivation (weakest correlation coefficient = −0.53, Supplementary Table 5), as well as with cognitive tests not used to derive *g* (weakest correlation co-efficient = 0.41, Supplementary Table 5). All correlations of *g* and individual cognitive tests were in the expected direction, where higher *g* correlated with higher score on each test (we note that negative correlation coefficients are expected when lower scores for a given test indicate higher cognitive ability). These results suggest *g* forms a cross-domain measure of generalised cognition.

We performed a sensitivity analysis of *g* by modifying some of the individual cognitive tests included in its formation (see Supplementary Materials 1.4 for details) and found alternative estimates of *g* were highly correlated with the original estimate (weakest correlation co-efficient = 0.8, Supplementary Table 7), suggesting *g* is robust to different input measures. We also note that *g* was significantly lower in UKBB participants with a diagnosis of schizophrenia, ASD or ID compared with participants without one of these disorders (β = –0.42, *p* = 1.9 × 10⁻⁶; Supplementary Figure 4). This supports the exclusion of participants with these disorders from our analysis of *g* to ensure our findings are not biased by individuals whose reduction in *g* may be due to the disorder itself and/or its treatment.

### Rare coding variants in schizophrenia-associated genes and generalised cognition

We investigated the relationship between damaging RCVs in schizophrenia-associated genes (see Methods and Supplementary Table 3 for a description of the gene sets tested) and *g* in UKBB participants without a diagnosis of schizophrenia, ASD, or ID. Supplementary Figure 5 presents a summary of analyses.

#### Constrained and brain expressed genes

**Fig. 1:**
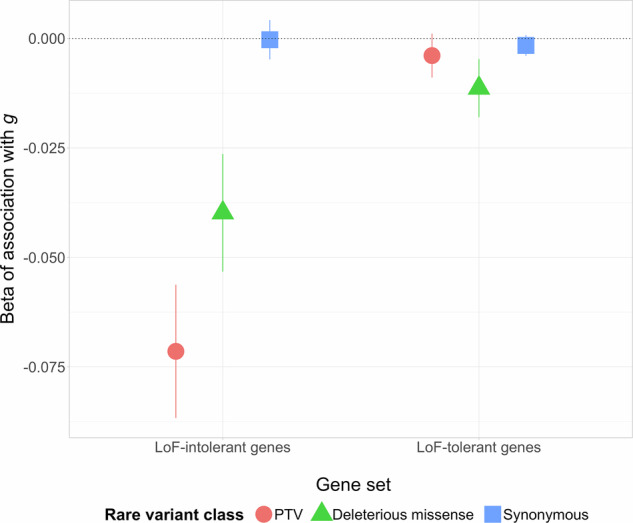
Association between *g* and different classes of rare coding variants in LoF-intolerant and LoF-tolerant genes in participants of 1KGP-EUR-like genetic ancestries. Error bars display 95% confidence intervals. LoF = loss-of-function; PTV = protein-truncating variant; *g* = generalised cognition.

In UKBB participants that were genetically similar to the 1KGP European superpopulation and who had sufficient cognitive test data for *g* to be derived (*n* = 75,188), rare PTVs and deleterious missense variants in LoFi genes were associated with lower *g* (rare PTVs: β = −0.071, *p* = 3.6 × 10^−20^; rare deleterious missense variants: β = −0.040, *p* = 6.3 × 10^−9^; Fig. 1; Supplementary Table 8). Rare synonymous variants in LoFi genes were not associated with *g* (Fig. 1; Supplementary Table 8). 15.8% of the sample carried at least one rare PTV in a LoFi gene, and 20.5% carried at least one rare deleterious missense variant in a LoFi gene (Supplementary Table 9). A greater number of rare PTVs and deleterious missense variants carried in LoFi genes was associated with lower *g* (Supplementary Figure 6). Similar effects on *g* were observed for RCVs in LoFi genes in males and females (Supplementary Figure 7). When less rare PTVs and deleterious missense variants (5 < MAC ≤ 50) in LoFi genes were tested, no significant associations with *g* were observed (Supplementary Figure 8). For genetic ancestries other than the 1KGP-EUR-like genetic ancestry group, no significant effects on *g* were observed for any class of RCV in LoFi genes (Supplementary Table 10), most likely due to small sample sizes. Therefore, all analyses presented hereafter are based on participants that are genetically similar to the 1KGP European superpopulation.

A multivariable linear regression model was used to test whether the effects of damaging RCVs in LoFi genes on cognition were independent of schizophrenia PRS and rare DD CNVs known to be enriched in schizophrenia. In 75,111 individuals of 1KGP-EUR-like ancestries in whom *g* could be derived and who had high-quality data for all classes of genetic variation investigated (RCVs, schizophrenia PRS, and rare DD CNVs known to be enriched in schizophrenia), we observed largely independent effects on *g*; the associations of *g* with rare PTVs and rare deleterious missense variants in LoFi genes remained significant when schizophrenia PRS and rare DD CNVs known to be enriched in schizophrenia were included in the model (Supplementary Table 11). This suggests that the effects on cognition of each of these classes of genetic variation are largely independent from one another.

Rare PTVs and rare deleterious missense variants in brain expressed genes (as defined in Supplementary Table 3) were associated with lower *g* (PTVs: β = −0.019, *p* = 6.0 × 10^−9^; deleterious missense variants: β = −0.020, *p* = 1.1 × 10^−6^; Supplementary Table 8). Rare PTVs in LoFi genes that were brain expressed had significantly stronger effects on *g* than LoFi genes that were not (z-test *p* = 0.0033). There was no significant difference in the effects on *g* between rare deleterious missense variants in brain expressed and non-brain expressed LoFi genes (z-test *p* = 0.47).

Rare PTVs and synonymous variants in LoF-tolerant genes were not associated with *g* (Fig. 1; Supplementary Table 8). Rare deleterious missense variants in LoF-tolerant genes were associated with lower *g* (β = −0.011, *p* = 0.0008; Fig. 1; Supplementary Table 8), although the effect size for these variants was significantly weaker than that observed for deleterious missense variants in LoFi genes (z-test *p* value = 9.8 × 10^−5^).

To assess whether RCVs in LoFi genes have effects on generalised cognitive ability beyond those captured by individual cognitive tests, we included individual cognitive test scores as covariates in the analysis of *g* with RCV burden in LoFi genes. Controlling for individual cognitive tests that were not included in the formation of *g* (trail-making test A, fluid intelligence, and symbol digit substitution), as well as those that were (numeric memory, reaction time, pairs matching, and trail making test B), only partly attenuated the significant association between rare PTVs and deleterious missense variants in LoFi genes and *g* (Supplementary Table 12). These findings suggest that damaging RCVs in LoFi genes have broad effects on cognition that extend beyond those captured by any individual cognitive test.

#### Genes associated with rare coding variants in schizophrenia

**Fig. 2:**
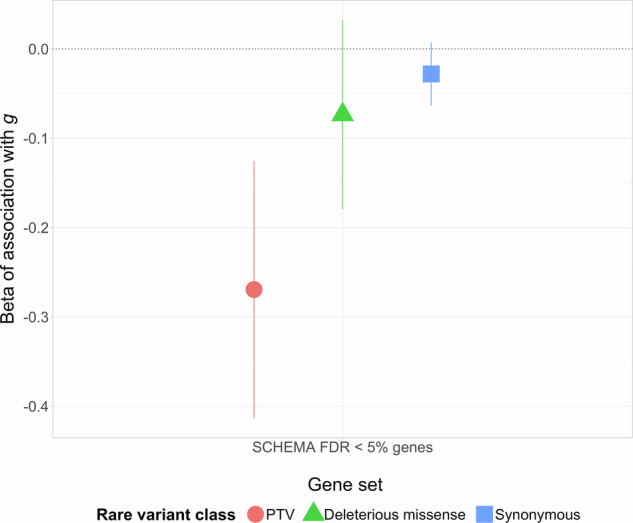
Association between *g* and different classes of rare variants in SCHEMA FDR < 5% genes from [28], in participants of 1KGP-EUR-like genetic ancestries. Error bars display 95% confidence intervals. FDR = false discovery rate; PTV = protein-truncating variant; g = generalised cognition.

We defined genes associated with RCVs in schizophrenia as those implicated in the disorder at a FDR < 5% in the SCHEMA study [28] (n autosomal genes = 29). Rare PTVs in this set were associated with lower *g* (β = −0.27, *p* = 0.0002) but rare deleterious missense were not (Fig. 2, Supplementary Table 13). PTVs in SCHEMA FDR < 5% genes were associated with *g* with a similar beta (β = −0.27) to that of rare DD CNVs known to be enriched in schizophrenia (β = −0.28, Supplementary Table 11). The effect on *g* for rare PTVs in SCHEMA FDR < 5% genes was significantly greater than that observed for rare PTVs in all LoFi genes and brain expressed genes (respective z-test p-values = 0.0034 and 0.0003).

#### Schizophrenia common allele loci

The largest schizophrenia GWAS of common variation to date [25] identified 282 associated autosomal loci and prioritised credible causal protein-coding genes for some of these loci. We analysed RCVs in the following schizophrenia common allele loci gene sets: 1) a broad set including all genes overlapping the common variant loci (*n* = 1715); 2) genes closest to the index SNP for each locus (*n* = 186); 3) and genes prioritised as credible causal in that paper [25] (*n* = 101).

**Fig. 3:**
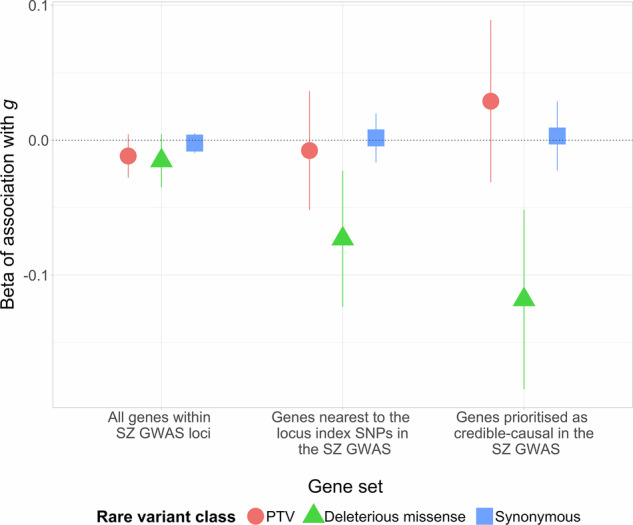
Association between *g* and different classes of rare variants in various schizophrenia (SZ) common allele loci/ GWAS sets, from [25], in participants of 1KGP-EUR-like genetic ancestries. Error bars display 95% confidence intervals. PTV = protein-truncating variant; g = generalised cognition.

Neither rare PTVs nor rare deleterious missense variants in genes mapping to broad schizophrenia common variant loci were associated with *g* (Fig. 3, Supplementary Table 14). At genes nearest the locus index SNP, rare deleterious missense variants (β = −0.073, *p* = 0.0045) but not rare PTVs were associated with *g* (Supplementary Table 14). The same was true for the credible causal genes (rare deleterious missense variants β = −0.12, *p* = 0.0005; Fig. 3; Supplementary Table 14). The effect on *g* of rare deleterious missense variants in credible causal genes was significantly greater than in non-prioritised genes at GWAS loci (z-test *p* = 0.0008). It was also greater than the effect of rare deleterious missense variants in LoFi genes in general (z-test *p* = 0.010), which is important to demonstrate since schizophrenia common alleles are enriched for LoFi genes [24].

Within the credible causal set, rare deleterious missense variants in the 61 fine-map prioritised genes (β = −0.12, *p* = 0.0029) and in the 44 SMR prioritised genes (4 of which were also prioritised by fine-mapping, β = −0.12, *p* = 0.046) had similar effect sizes (Supplementary Figure 9; Supplementary Table 14). Prioritisation as a credible causal candidate by SMR [25] required a gene to have a brain eQTL and therefore brain expression could act as a confounder. The effects on *g* for rare deleterious missense variants in credible causal genes, and also in the subset of SMR prioritised genes, were significantly stronger than in all brain-expressed genes that were not part of these respective sets (credible causal genes vs. brain-expressed genes: z-test *p* = 0.0018; SMR prioritised genes vs. brain-expressed genes: z-test *p* = 0.048). Thus, the associations we observe here do not simply reflect a background association between *g* and rare deleterious missense variants in brain-expressed genes. We also explored whether the association between rare deleterious missense variants in the SMR prioritised genes was driven by genes linked to over- or under-expression in schizophrenia (see Supplementary Table 3 for definitions of these sets). Rare deleterious missense variants in the 20 genes associated with reduced expression in schizophrenia were significantly associated with lower *g* (β = −0.20, *p* = 0.014), whereas no effects were observed for rare deleterious missense variants in the 24 genes associated with increased expression in schizophrenia (β = 0.006, *p* = 0.94).

#### Schizophrenia CNV loci

RCVs in genes mapping to schizophrenia-enriched CNVs (n genes = 886) and schizophrenia-associated CNVs (n genes = 178) were tested for association with *g* (see Methods and Supplementary Table 3 for further details on these CNV gene sets).

No class of RCV in the 886 genes mapping to schizophrenia-enriched CNVs was associated with *g* (Supplementary Table 15). Rare PTVs in LoFi genes within the schizophrenia-enriched CNV loci (n genes = 144) were associated with lower *g* (β = −0.11, *p* = 0.0026, Supplementary Table 15) but the effect size did not significantly differ from that in LoFi genes outside CNV loci (z-test *p* = 0.15). No class of RCV in LoF-tolerant genes within the schizophrenia-enriched CNV loci was associated with *g* (Supplementary Table 15).

No associations with *g* were observed for any class of RCV within the 178 genes mapping to the schizophrenia-associated gene set or in the subsets of those genes that were LoFi or LoF-tolerant (Supplementary Table 15).

### Single gene burden tests

Single-gene association tests were performed for three classes of RCVs (PTVs, deleterious missense variants, and a combined (PTV + deleterious missense) variant class) in 1003 unique genes taken from the SCHEMA FDR < 5% gene set, the credible causal schizophrenia GWAS gene set, and genes within schizophrenia-enriched CNV loci (number of single gene tests = 3009). We did not test genes with fewer than five qualifying variants due to a lack of power. No gene showed significant evidence for association after Bonferroni correction (corrected p value 0.05/3009 = 1.66 × 10^−5^; Supplementary Table 16).

## Discussion

In the UKBB, we show that damaging RCVs in genes and loci implicated in schizophrenia by common variant and RCV studies are associated with lower *g* in participants without a diagnosis of schizophrenia, ASD or ID. To our knowledge, this is the first study to investigate the relationship between RCVs in schizophrenia genes and *g*. We focussed on *g*, as opposed to individual cognitive tests, for two main reasons. First, a large body of evidence suggests schizophrenia is associated with global cognitive impairment [8, 15, 16], and compared to controls, people with schizophrenia show greater impairment in *g* than for tests of specific cognitive domains [72]. Second, compared to individual cognitive tests, *g* reduces noise by limiting test-specific error and is a robust measure of general cognitive ability [10, 11, 69].

Genes under selective constraint for PTVs have consistently been shown to be enriched for common alleles, rare CNVs, and RCVs in people with schizophrenia [22–24]. Here, we provide novel lines of evidence demonstrating that damaging RCVs in these LoFi genes also contribute to *g* in people without a diagnosis of schizophrenia, ASD, or ID. We first show that rare PTVs and deleterious missense variants in LoFi genes are significantly associated with lower *g*, and that these associations remained significant when adjusting for carriers of rare DD CNVs known to be enriched in schizophrenia and schizophrenia PRS, both of which are associated with cognition in unaffected individuals [30, 40, 73]. This suggests the impact of RCVs on *g* is at least partially independent of these other genetic factors. We then show that the association of *g* with RCVs in LoFi genes is not explained by any individual cognitive test, including reaction time and fluid intelligence, both of which have recently been associated with damaging types of RCVs in LoFi genes [32, 36]. These findings suggest the impact of these variants extends beyond any individual cognitive domain, and that they have a broad, generalised effect on cognitive function. Such a generalised effect of these RCVs suggests they influence brain mechanisms or systems that control processes central to cross-domain cognition.

RCVs in LoFi genes are associated with both schizophrenia and cognition, but there are many LoFi genes and whether the same LoFi genes have pleiotropic effects on schizophrenia and cognition in unaffected individuals is unknown. We show for the first time that carriers of rare PTVs in genes associated with RCVs in schizophrenia have significantly lower *g*, and that the effect of rare PTVs in SCHEMA FDR < 5% genes on *g* is significantly greater than that observed for rare PTVs in LoFi genes and in brain expressed genes. This finding is consistent with genic pleiotropy between schizophrenia and general cognitive function in unaffected individuals. We note that carriers of rare PTVs in SCHEMA FDR < 5% genes had a 0.27 standard deviation decrease in *g*, which is similar to the reduction in *g* observed for carriers of rare DD CNVs known to be enriched in schizophrenia (0.28 standard deviations).

We also provide novel evidence that rare deleterious missense variants in credible causal genes within schizophrenia-associated common variant loci are associated with lower *g*, and that this is not confounded by the fact that this set of genes is enriched for LoFi genes and those that are brain expressed. These findings advance previous work that only examined the effects on cognitive traits of RCVs in broadly defined schizophrenia common allele loci, and did not control for the effects of constraint or brain expression [32]. To gain mechanistic insight into the association between rare deleterious missense variants in credible causal genes and *g*, we stratified the SMR-prioritised genes into those over- and under-expressed in schizophrenia. Significant effects on *g* were only observed for rare deleterious missense variants in genes linked to under-expression in schizophrenia. This suggests that haploinsufficiency, at least in part, underlies the association between missense variants in schizophrenia credible causal genes and lower *g*. Moreover, our analysis of schizophrenia credible causal genes provides orthogonal support for the SMR and fine-mapping approaches used to prioritise credible causal genes for schizophrenia in the study by Trubetskoy et al. [25], indicating that data from population studies of RCVs could assist gene prioritisation within GWAS loci.

CNVs associated with developmental disorders are collectively enriched in schizophrenia [26] and associated with lower cognitive test scores in unaffected individuals [30, 31]. Here, we mirror findings from other cohorts demonstrating lower generalised cognition in carriers of these rare DD CNVs known to be enriched in schizophrenia [74]. We also found that rare PTVs in LoFi genes overlapping the CNV loci are associated with lower *g*, however, these effects were not significantly different from those observed for rare PTVs in all LoFi genes, contrasting with our findings based on other schizophrenia gene sets. We speculate that this reflects the highly polygenic nature of schizophrenia [75], and the multigenic nature of all but one schizophrenia-associated CNV, a consequence of which would be that unless each CNV contains a large number of schizophrenia-causal genes, the proportion of such genes in CNVs is unlikely to be substantially elevated over LoFi genes in general.

By finding the strongest effects on *g* for RCVs in genes that have individually been implicated in schizophrenia (i.e. the credible causal genes and RCV associated gene sets), our study supports the hypothesis that the biology of schizophrenia overlaps that for general cognitive function in the population. These sets of genes also have plausible biological links to cognition, given they are enriched for genes encoding proteins related to the synapse [25, 28], which has important roles in learning and memory [76]. However, our findings do not address the question of whether the effects of rare variants on risk of schizophrenia are mediated by their effects on cognitive function, highlighting an important topic for future work.

The favourable psychometric properties of *g* compared to individual cognitive tests [10, 11] is a strength of our study, but the associated requirement for multiple cognitive test scores will almost certainly induce a participation bias that would lead to under-representation of mutations that reduce the likelihood of completing all cognitive tests. This is plausible since previous work has shown that UKBB participants with PTVs in genes associated with ASD or in LoFi genes are less likely to have completed cognitive testing [77]. The known UKBB volunteer bias, whereby participants have a higher average socio-economic status and are generally healthier than the UK population [78] is likely to have a similar effect. Both biases would result in underestimation of the effect sizes of variants that impact *g*, but are unlikely to change the general direction of observed effects [79]. Future research focused on understanding the relationship between different classes of RCVs and participation in the voluntary and cognitive components of the UKBB could inform the interpretation of findings from this and wider studies.

One limitation of examining cognition and schizophrenia-associated genetic variation in this cohort is that the UKBB cognitive test battery does not examine all domains of cognition impacted in schizophrenia (for instance as defined by the MATRICS initiative [80]). In the UKBB, the only tests examining verbal learning and higher-level executive function are completed by relatively few individuals, and there is no formal assessment of social cognition. Importantly, previous work has shown that *g* derived from UKBB tests correlates strongly with *g* derived from more comprehensive cognitive test batteries [69], and thus our use of *g* offers a route to examine broad, cross domain cognition despite the limitations of the individual cognitive measures in the UKBB. Furthermore, as population biobank sample sizes increase, sample sizes with scores for individual cognitive tests will also increase. It will then become possible to examine the effects of damaging RCVs in schizophrenia genes on cognitive domains with particular relevance to schizophrenia, beyond effects on *g*, which may provide additional biological insights.

While our findings show that damaging RCVs in schizophrenia-associated genes are associated with lower *g* in the general population, the effect sizes we observe may differ in clinical populations, as we studied participants without a diagnosis of schizophrenia, ASD, or ID to reduce the likelihood of our findings reflecting reverse causation. Another limitation this study faced, despite the large sample size, was that some analyses were underpowered; for example, we lacked power to implicate individual genes in cognition. We were also underpowered to analyse RCVs in non-1KGP-EUR-like genetic ancestries. It is important for future work to examine the relationship between damaging RCVs in schizophrenia-associated genes and loci and cognition in clinical populations and in more diverse and representative samples.

In conclusion, we show for the first time that damaging RCVs in LoFi genes contribute to variation in generalised cognition in UKBB participants without a diagnosis of schizophrenia, ASD, or ID, with significantly stronger effects on cognition observed for damaging RCVs in genes previously associated with schizophrenia. These findings suggest that biology impacted in schizophrenia by common and rare alleles is associated with cognition in the population, and that the genetic overlap between schizophrenia and cognition in unaffected individuals is not explained by general gene properties such as LoF-intolerance or brain expression. They also suggest that the association of impaired cognition in schizophrenia is, at least in part, explained by shared biology between schizophrenia and cognitive function. Our study demonstrates the utility of exploiting large sequencing datasets of unaffected individuals, such as the UKBB, to identify genes with shared effects on cognition and schizophrenia and provides a route towards determining the biological processes underlying cognitive impairment in the disorder.

## Supplementary information

Supplementary Materials

Supplementary Tables

## Data Availability

This research was conducted using the UK Biobank Resource under Application Number 13310. Data from the UK Biobank is available for health-related research upon registration and application through the UK Biobank Access Management System (https://www.ukbiobank.ac.uk/enable-your-research/register). UK Biobank whole exome sequencing data in pVCF format was accessed using data-field ID 23157. The code required to reproduce our analyses is publicly available (https://github.com/eilidhfenner/UKBB_RAP_WES).

## Code availability

The code required to reproduce our analyses is publicly available (https://github.com/eilidhfenner/UKBB_RAP_WES). Table 1 lists all software packages and tools used in the analyses presented in this manuscript. All of these are publicly available and where applicable, versions and source URLs are provided in the table.

**Table 1:** Key resources table presenting sources of software used in this manuscript.

RESOURCE	SOURCE	IDENTIFIER
Hail (v0.2.60) running on a JupyterLab instance on the UKBB research analysis platform (RAP)	Hail team [49]	https://github.com/hail-is/hail
R (v4.4.0) in RStudio (v2.2.1) running on a Posit Workbench on the UKBB RAP	R core team and Posit	https://cran.r-project.org/ and https://posit.co/
PLINK (v1.9 and v2.0)	Chang et al. [81]	https://www.cog-genomics.org/plink/1.9/ and https://www.cog-genomics.org/plink/2.0/
PRSice-2	Choi & O’Reilly [37, 82]	https://choishingwan.github.io/PRSice/
Ensembl’s VEP running in Hail using LoFTEE plugin	VEP: McLaren et al. [62] LoFTEE: Karczewski et al. [63, 64]	https://hail.is/docs/0.2/methods/genetics.html#hail.methods.vep and https://github.com/konradjk/loftee
r/ggplot2 (v3.5.1)	Wickham [83]	https://ggplot2.tidyverse.org/
r/ukbtools (v0.11.3)	Handscombe et al. [51]	https://kenhanscombe.github.io/ukbtools/
r/data.table (v1.15.4)	Barrett et al. [84]	https://r-datatable.com
r/dplyr (v1.1.4)	Wickham et al. [85]	https://dplyr.tidyverse.org
r/broom (v1.0.8)	Robinson et al. [86]	https://broom.tidymodels.org/
Scripts used for analysis in this study	This study	https://github.com/eilidhfenner/UKBB_RAP_WES
